# Advanced maternal age: copy number variations and pregnancy outcomes

**DOI:** 10.3389/fgene.2023.1206855

**Published:** 2023-06-15

**Authors:** Luoyuan Cao, Wenxu Dong, Qinjuan Wu, Xiaomin Huang, Xiaomei Zeng, Jing Yang, Jiaojiao Lu, Xunyan Chen, Xian Zheng, Xianguo Fu

**Affiliations:** ^1^Department of Central Laboratory, Ningde Municipal Hospital Affilliated to Ningde Normal University, Ningde, Fujian, China; ^2^Department of Obstetrics, Ningde Municipal Hospital Affilliated to Ningde Normal University, Ningde, Fujian, China; ^3^Department of Ultrasound, Ningde Municipal Hospital Affilliated to Ningde Normal University, Ningde, Fujian, China

**Keywords:** advanced maternal age, prenatal diagnosis, copy number variations, karyotyping, pregnancy outcomes

## Abstract

**Objective:** Adverse pregnancy outcomes are closely related to advanced maternal age (AMA; age at pregnancy ≥35 years). Little research has been reported on aneuploid abnormalities and pathogenic copy number variations (CNVs) affecting pregnancy outcomes in women with AMA. The purpose of this study was to assess CNVs associated with AMA in prenatal diagnosis to determine the characteristics of pathogenic CNVs and assist with genetic counseling of women with AMA.

**Methods:** Among 277 fetuses of women with AMA, 218 (78.7%) were isolated AMA fetuses and 59 (21.3%) were non-isolated AMA fetuses and showed ultrasound anomalies from January 2021 to October 2022. Isolated AMA was defined as AMA cases without sonographic abnormalities. Non-isolated AMA was defined as AMA cases with sonographic abnormalities such as sonographic soft markers, widening of the lateral ventricles, or extracardiac structural anomalies. The amniotic fluid cells underwent routine karyotyping followed by single nucleotide polymorphism array (SNP-array) analysis.

**Results:** Of the 277 AMA cases, karyotype analysis identified 20 chromosomal abnormalities. As well as 12 cases of chromosomal abnormalities corresponded to routine karyotyping, the SNP array identified an additional 14 cases of CNVs with normal karyotyping results. There were five pathogenetic CNVs, seven variations of uncertain clinical significance (VOUS), and two benign CNVs. The detection rate of abnormal CNVs in non-isolated AMA cases was increasing (13/59; 22%) than in isolated AMA cases (13/218; 5.96%) (*p <* 0.001). We also determined that pathogenic CNVs affected the rate of pregnancy termination in women with AMA.

**Conclusion:** Aneuploid abnormalities and pathogenic CNVs affect pregnancy outcomes in women with AMA. SNP array had a higher detection rate of genetic variation than did karyotyping and is an important supplement to karyotype analysis, which enables better informed clinical consultation and clinical decision-making.

## Introduction

Pregnancies in women of AMA are speedily growing and are related to aneuploidy, copy number variations (CNVs), aberrant trophoblast cell function, cardiovascular health, and unfavorable pregnancy outcomes (Cooke and Davidge, 2019; Pinheiro et al., 2019; Frick, 2021; Xiong et al., 2021). A relation between maternal age and chromosomal aneuploidy has been established in previous studies. However, few studies exist on the association between AMA and CNV. The risk of sex chromosomal aneuploidy or autosomal aneuploidy rises significantly with maternal age, especially after 35 years. One previous study suggested that AMA is an independent indicator of placental pathologic examination (Torous and Roberts, 2020). Owing to aging of the oocytes perse in women with AMA, the errors of chromosome segregation during meiotic division are becoming more common, and easy to the production of oocytes with an incorrect number of chromosomes, resulting in an increased risk of fetal chromosomal aneuploidy (Mikwar et al., 2020; Wasielak-Politowska and Kordowitzki, 2022). Maternal age negatively affects imprinted gene methylation and expression in both the germ and somatic cells of the reproductive tract, contributing to reduced fertility in women with AMA (Paczkowski et al., 2015). Interestingly, post-translational modifications in ovarian oocytes in pregnant women lead to fetal chromosomal abnormalities (Van den berg et al., 2011; Ling et al., 2018; He et al., 2019). However, the frequency of abnormal CNV in offspring was not related to maternal age, and the risk of abnormal CNV remained constant during the reproductive period of a women (Sibbons et al., 2012). Most of the previous studies have suggested that CNVs are closely associated with maternal age; in particular, maternal age appears to increase the risk of the occurrence of *de novo* non-complex CNVs in progeny with intellectual disabilities or delayed developmental, however, there is no evidence that increasing paternal age has an effect on *de novo* CNV formation (Ma et al., 2020; Wadhawan et al., 2020). Thus, factors involved in AMA must be identified for improved diagnosis and treatment of this condition.

Chromosome microarray analysis (CMA), also known as molecular karyotype analysis, uses gene-chip technology to identify chromosomal diseases during prenatal diagnosis (Levy and Wapner, 2018; Wu et al., 2021). CMA technology is a high-resolution method for genome-wide detection and has been recommended as a primary testing for prenatal diagnosis. This method can be used to detect CNVs of chromosome imbalances, such as aneuploidy and unbalanced rearrangements, especially chromosome microdeletion and duplication (Hsiao et al., 2022; Li et al., 2022; Liu et al., 2022). CMA includes microarray-based comparative genomic hybridization and SNP array. SNP array would detect uniparental disomy (UPD), loss of heterozygosity (LOH), and low-level mosaicism. In this study, we performed whole-genome scanning using an SNP array and routine karyotyping of 277 fetuses from women with AMA. We attempted to elucidate chromosomal abnormalities and CNVs in these fetuses to explore the clinical value of applying an SNP array in women with AMA.

The purpose of this study was to investigate the characteristics of chromosomal abnormalities and pathogenic CNVs in women with AMA. Provides the basis for prenatal genetic counselling, in order to reduce the harmful effects and reduce its occurrence, is very important to prevent abnormal reproductive outcomes in the population.

## Materials and methods

### Patient data

We conducted a retrospective study on women with AMA diagnosed at prenatal diagnosis institutions of the Ningde Municipal Hospital (affiliated with the Ningde Normal University) between January 2021 and October 2022. Fetal samples were collected using amniocentesis (*n* = 277) at the gestational age of 18–26 weeks. The fetuses underwent a routine ultrasonic examination, and fetal biometry was implemented at a median gestational age of 20^+4^ weeks (range, 18^+1^ to 26^+2^ weeks). The median age at pregnancy was 38 years (range, 35–47 years). This study was approved by the ethics committee of the Ningde Municipal Hospital (affiliated with the Ningde Normal University) (Clearance No.: NSYKYLL-2023-028), and informed consent were obtained from all participants for amniocentesis. Once pathogenic CNVs of fetal was confirmed, about 2.0 mL of peripheral venous blood was collected from the parents to verify inheritance. DNA was extracted using a Gentra Puregene Blood Kit (QIAGEN, Santa Clara, CA, United States).

Cases were grouped as isolated AMA and non-isolated AMA; isolated AMA was defined as AMA cases without sonographic abnormalities. Non-isolated AMA was defined as AMA cases with sonographic abnormalities such as sonographic soft markers, widening of the lateral ventricles, or extracardiac structural anomalies. A total of 277 AMA cases were included; 218 cases (78.7%) were isolated AMAs and 59 cases (21.3%) were non-isolated AMAs (Table 1).

**TABLE 1:** Phenotypic characteristics of 277 AMA fetuses.

Classification	Number of fetuses	Number of karyotype	Number of SNP-array		
			Pathogenic CNVs	VOUS	Benign CNVs
Isolated-AMA	218	15	4	7	2
Non-isolated-AMA	59	5	13	0	0
Total	277	20	17	7	2

AMA, advanced maternal age; SNP-array, single nucleotide polymorphism array; CNVs, copy number variation; VOUS, variation of uncertain clinical significance.

### Cytogenetic analysis

Amniocytes and lymphocytes were analyzed through routine karyotyping using the Giemsa banding technique at a band resolution of 400–550.

### Chromosomal microarray analysis (SNP-array)

According to the manufacturer’s instructions, used the QIAamp^®^ DNA Blood kits (Qiagen, Valencia, CA, United States) to directly extract fetal genomic DNA from uncultured amniotic fluid. DNA was quantified using a NanoDrop spectrophotometer (Thermo Fisher Scientific, Waltham, MA,United States), according to the manufacturer’s instructions. In brief, fetal DNA was digested, attached to adapters, amplified, purified, fragmented, labeled with biotin, and then hybridized using an Affymetrix^®^ CytoscanTM 750 K array system (Affymetrix, Santa Clara,CA, United States). This system contains SNP and CNV probes and can detect CNVs, mosaic (mosaic proportion >10%), and LOH. The CNV reporting filter was set to >100 kb, with a minimum set of 50 marker counts. The results obtained from the CytoScan arrays were analyzed using the Chromosome Analysis Suite software (Affymetrix) with annotations of the genome version GRCH37 (hg19). The detected copy number gains or losses were systematically evaluated by comparison with values available in the literature and publicly available databases, including the Database of Genomic Variants (http://projects.tcag.ca/variation/), the DECIPHER database (http://decipher.sanger.ac=/), the International Standards for Cytogenomic Arrays (https://www.iscaconsortium.org/), and Online Mendelian Inheritance in Man (OMIM; http://www.omim.org). CNVs were divided into three categories: benign, pathogenic, or variants of uncertain significance (VOUS), according to the American College of Medical Genetics guidelines (Riggs et al., 2020; Riggs et al., 2021, Correction). SNP-array analysis of DNA from maternal and paternal blood samples was performed to determine whether the CNVs detected in the fetal samples were *de novo* or inherited. All CNVs of *de novo* were validated using fluorescence *in situ* hybridization (FISH).

### Statistical analysis

IBM SPSS Statistics version 20 (IBM Corp., Armonk, NY, United States) was used for statistical analysis. The detection rate of pathogenic CNVs in non-isolated and isolated AMA fetuses and ultrasound anomalies were determined using the chi-square (χ^2^) test. The difference was statistically significant, setting at *p* < 0.05.

## Results

A total of 277 AMA cases were included, of which 218 (78.7%) were isolated and 59 (21.3%) were non-isolated (Table 1). We identified abnormal CNVs in 26 (9.38%) cases of AMA; of these, 17 were pathogenic, seven were VOUS, and two were benign. The detection rate of abnormal CNVs in the non-isolated AMA group was higher (22%; 13/59) than that in the isolated AMA group (5.96%; 13/218) (*p <* 0.001).

### Cytogenetic analysis of AMA fetuses

Karyotyping identified 20 chromosomal abnormalities in the 277 AMA cases analyzed. Among the 277 fetuses in the pregnant woman with AMA, karyotyping identified 17 chromosomal abnormalities with clinically significant, including trisomy 18 (Trisomy 18 Syndrome) (*n* = 2) and trisomy 21 (Down Syndrome) (*n* = 10), in accordance with the results of the SNP-array. Five cases showed abnormal chromosome structures, such as pericentric (9) (p12q13) inversion (*n* = 3), one case had pericentric (Y) (p11.2q11.2) pat inversion, one case showed balanced genetic translocation t (4;11) (p13;q21), and three cases exhibited chromosome polymorphisms associated with 14pss, 15cenh+, and 1qh+ (Table 2).

**TABLE 2:** Abnormal karyotyping results of 277 AMA fetuses.

Case	Karyotype	SNP-array results	Postnatal outcome
1	47,XY,+18	arr [hg19](18) × 3	TP
2	47,XX,+18	arr [hg19](18) × 3	TP
3	47,XX,+21	arr [hg19](21) × 3	TP
4	47,XX,+21	arr [hg19](21) × 3	TP
5	47,XX,+21	arr [hg19](21) × 3	TP
6	47,XY,+21	arr [hg19](21) × 3	TP
7	47,XX,+21	arr [hg19](21) × 3	TP
8	47,XX,+21	arr [hg19](21) × 3	TP
9	47,XY,+21	arr [hg19](21) × 3	TP
10	47,XX,+21	arr [hg19](21) × 3	TP
11	47,XY,+21	arr [hg19](21) × 3	TP
12	47,XY,+21	arr [hg19](21) × 3	TP
13	46,XX,14pss	arr [hg19](1–22) × 2, (XX) × 1	TD
14	46,XY,inv (9) (p12q13)	arr [hg19](1–22) × 2, (XY) × 1	TD
15	46,XX,t (4;11) (p13;q21)	arr [hg19](1–22) × 2, (XX) × 1	TD
16	46,X,inv(Y) (p11.2q11.2)pat	arr [hg19](1–22) × 2, (XY) × 1	TD
17	46,XX,inv (9) (p12q13)	arr [hg19](1–22) × 2, (XX) × 1	TD
18	46,XX,inv (9) (p12q13)	arr [hg19](1–22) × 2, (XX) × 1	TD
19	46,XY,15cenh+	arr [hg19](1–22) × 2, (XY) × 1	TD
20	46,XX,1qh+	arr [hg19](1–22) × 2, (XX) × 1	TD

AMA, advanced maternal age; SNP-array, single nucleotide polymorphism array; TP, termination of pregnancy; TD, term delivery.

### SNP array results

In addition to the 12 fetuses with chromosomal abnormalities in accordance with the results of routine karyotyping, the SNP-array identified an additional 14 cases (5.1%) of abnormal CNVs. Of these, five were pathogenic CNVs, two were benign, and seven were VOUS. Abnormal CNVs ranged 0.33–34.3 Mb in size. Among the five pathogenic CNVs, three showed microdeletion/microduplication syndrome (22q11.2 microdeletion syndrome, 16p11.2 microdeletion syndrome, and 13q31.3 microduplication) and two showed mosaic trisomy 22 or 15q11.2q15.1 LOH. Seven fetuses exhibited VOUS CNVs, including four with duplications and three with LOH. VOUS CNVs were associated with duplications of 2p12, 8q24.3, 16p13.2, and Xp22.31 and LOH of 1p31.3p31.1,3q23q25.1, 7p12.3p11.1 and 12q21.31q21.33, and two fetuses were associated with microduplication of 1q21.1 or 22q11.23 inherited paternally (Table 3). The chromosome polymorphisms did not show abnormalities in the SNP-array, such as 14pss, 15cenh+, and 1qh+.

**TABLE 3:** SNP array analysis of AMA fetuses with normal karyotyping results.

Case	CMA results	Size (Mb)	Inheritance	Pathogenicity classification	Postnatal outcome
1	arr [hg19]22q11.21 (18,648,856-21,800,471) × 1	3.1	*De novo*	P (DiGeorge syndrome)	TP
2	arr [hg19]16p11.2 (29,580,021-30,176,508) × 1	0.58	*De novo*	P (16p11.2 deletion syndrome)	TP
3	arr [hg19]13q31.3 (92,446,505-93,978,999) × 3	1.5	*De novo*	P(FRG)	TP
4	arr [hg197](22) × 2-3	34.3	*De novo*	P (mosaic trisomy 22)	TP
5	arr [hg19]15q11.2q15.1 (22,817,871-40,784,285) × 2hmz	17.9	*De novo*	P(Prader-Willi syndrome)	TP
6	arr [hg19]2p12 (78,631,710-79,851,089) × 3	1.2	*De novo*	VOUS	TD
7	arr [hg19]8q24.3 (141,675,931-142,994,223) × 3	1.3	*De novo*	VOUS	TD
8	arr [hg19]16p13.2 (8978102_9321033) × 3	0.33	*De novo*	VOUS	TD
9	arr [hg19]Xp22.31 (6440780_8135568) × 3	1.6	*De novo*	VOUS	TD
10	arr [hg19]1p31.3p31.1 (65,703,484-77,130,322) × 2hmz	11.4	*De novo*	VOUS	TD
11	arr [GRCh37]3q23q25.1 (139589227_151956589) × 2hmz	12.3	*De novo*	VOUS	TD
12	arr [hg19]7p12.3p11.1 (47,433,733-58,019,983) × 2hmz, 12q21.31q21.33 (80,884,523-92,341,219) × 2hmz	10.5	*De novo*	VOUS	TD
		11.4			
13	arr [hg19]1q21.1 (145,382,124-145,988,238) × 3	0.59	Paternal	Benign	TD
14	arr [hg19]22q11.23 (23,653,980-25,041,592) × 3	1.3	Paternal	Benign	TD

AMA, advanced maternal age; CMA, chromosomal microarray analysis; TP, termination of pregnancy; TD, term delivery.

### Inheritance analysis

We verified the inheritance information of the 14 families with abnormal CNVs. The analysis showed that two fetuses inherited abnormal CNVs from unaffected parents, whereas 12 were *de novo* CNVs.

### Pregnancy outcomes and clinical follow-up

We obtained delivery information concerning 277 pregnancies in women with AMA, including 17 cases of termination of pregnancy (6.13%; 17/277); 12 had aneuploidies and 5 had pathogenic CNVs. SNP array analyses showed abnormalities in 14 fetuses with normal karyotyping results, among which 5 cases of pathogenic CNVs were found, including mosaic trisomy 22, 22q11.21 microdeletion, 13q31.3 microduplication, 16p11.2 microdeletion, and 15q11.2q15.1 LOH. Labor was induced in the mothers of the fetuses with pathogenic CNVs. Therefore, SNP array had a higher detection rate of genetic variation than did karyotyping and is an important supplement to karyotype analysis.

## Discussion

Karyotyping has been considered the gold standard for detecting chromosomal abnormalities for a long time in the past and can detect numerical abnormalities and large structural abnormalities of chromosomes (>5–10 Mb) during prenatal diagnosis (Wapner et al., 2012). However, because the SNP array can detect deletions, duplications, UPD, and low-level mosaicism at the submicroscopic level, it has replaced routine karyotyping as the first-tier test for prenatal diagnosis in some countries (Bedei et al., 2021). The advantage of karyotyping is that it can be used to diagnose balanced translocations, such as free trisomies, and unbalanced Robertsonian translocations. An inherited balanced rearrangement has no consequences for the current pregnancy; however, meiosis in germ cells with balanced translocations may result in meiotic arrest and unbalanced gametes or subsequent infertility, with attendant risks of miscarriage and unbalanced progeny (Wilch and Morton, 2018). Therefore, balanced translocation should be a guide for subsequent fertility counseling. Submicroscopic deletions or duplications are typically smaller than 5–10 Mb; they are often missed when conventional karyotyping is used. Previous studies have shown that microarray analysis yields 5%–9% additional clinically valuable information over conventional karyotyping (Grande et al., 2015; Levy and Wapner, 2018; Cai et al., 2020).

In this study, we found 12 cases of numerical chromosomal abnormalities, 5 of structural abnormalities, and 3 of normal variation using karyotyping of amniotic fluid cells from 277 pregnant women with AMA. The additional diagnostic yield was 14 cases (5.1%) of abnormal CNVs for fetuses with normal conventional karyotyping by SNP array, and 5 cases of pathogenic CNVs were included. Details of these five cases of pathogenic CNVs are as follows:

The SNP array results of case 1 (Table 3), the fetus had a 3.1 Mb deletion in the 22q11.21 region, which contained 49 OMIM genes, including *CLTCL1, HIRA,* and *TBX1*. Reportedly, the 22q11.2 deletion is associated with the 22q11.2 deletion syndrome. This syndrome, also known as DiGeorge syndrome or velocardiofacial syndrome, is autosomal dominant, and approximately 93% of the abnormalities are *de novo*. Approximately 7% of these abnormalities are inherited from the parents (McDonald-McGinn et al., 1999). This is therefore a *de novo* case of 22q11.2 deletion syndrome.

In case 2, the fetus had a 0.58 Mb deletion in the 16p11.2 region, which contained 22 OMIM genes, including *PRRT2, TBX6,* and *KCTD13.* A mutation in *TBX6* is associated with spondylocostal dysostosis, with clinical phenotypes including segmentation defect of vertebrae, irregular arrangement of ribs, and reduction of ribs. Previous studies have reported that such deletions are associated with the 16p11.2 deletion syndrome and various clinical phenotypes, including retardation, intellectual disability, autism spectrum disorders, and obesity (Chung et al., 2021). Another study reported that the *TBX6* deletion has been detected in patients with congenital scoliosis (Wu, et al., 2015). Previous studies have reported that the penetrance of 16p11.2 deletions was 46.8%, suggesting incomplete or differential penetrance (Rosenfeld et al., 2013).

In case 3, 13q31.3 with a 1.5 Mb segment duplication containing the *GPC6* gene was observed. Homozygous or compound heterozygous mutations in *GPC6* are associated with autosomal recessive omodysplasia. On the basis of the ultrasound findings, the fetus was diagnosed with growth retardation; its mother’s first child was diagnosed with growth retardation after birth. Therefore, the woman chose to terminate the current pregnancy (Trajanoska and Rivadeneira, 2019; Bayat et al., 2020).

In case 4 (Table 3) showed that the fetus had a low proportion of trisomy 22 mosaicism. Trisomy 22 is a common aneuploid abnormality that occurs in a high proportion of autosomal trisomies, accounting for approximately 3%–5% of abortions. Fully non-mosaic trisomy 22 is generally considered fatal, and only a small number of fetuses survive beyond 12 weeks. Depending on the proportion of abnormal cells, patients may present with different clinical phenotypes, including cleft lip and palate, oligohydramnios, intrauterine growth retardation, microcephaly, facial deformities, congenital heart defects, and urogenital malformations (Ma et al., 2018; Chen et al., 2019). The clinical phenotype and severity of the disease may vary according to the proportion of abnormal cells and their distribution in tissues. Therefore, in this case, the pregnant woman did not accept the risk of uncertainty and decided to terminate the pregnancy at 24^+6^ weeks.

In case 5, the SNP array results showed that the fetus had no abnormal CNVs in the entire chromosomal genome. However, the SNP array revealed a 17.9 Mb LOH on 15q11.2q15.1, including *MKRN3, MAGEL2, NDN, SNRPN, UBE3A,* and *ATP10C*, which are associated with gene imprinting. Previous studies have reported that 15q11.2q13 is the pathogenic imprinted gene region associated with Prader-Willi syndrome/Angelman syndrome (Del Gaudio et al., 2020). Therefore, the CNV was classified as pathogenic in case 5, and the pregnancy was terminated.

In this study, we found that fetuses with Down syndrome (DS, full trisomy of chromosome 21) had the highest proportion of pathogenic variants (58.8%; 10/17) among women of AMA. DS is a genetically complex condition compatible with post-term human survival and the most frequent survivable autosomal aneuploidy, with a common feature being intellectual disability (Coppede, 2016). Several studies have described the clinical characteristics of such pregnancies and showed that AMA at conception is a major risk factor for DS (Gruhn et al., 2019). Aging of the oocytes perse in pregnant women with AMA increases the risk of aneuploidy (Aprigio et al., 2022). However, there is no evidence of the influence of increased paternal age on *de novo* CNV formation (Wadhawan et al., 2020). We determined that AMA is a high-risk factor for autosomal aneuploidy and is also closely related to CNVs such as triploidy mosaicism, microduplication, microdeletion, and LOH. The pathogenic CNVs were not detected using karyotyping. Therefore, a SNP array is necessary and effective for identifying pathogenic CNVs in fetuses with AMA and can aid parental counseling decision-making. In addition, we should note that loss of fetuses with chromosomal abnormalities would affect the percentage of abnormality detection. Due to the amniocenteses were taken during a narrow window of trimester world-wide. Therefore, we will also focus on the genetic research of early miscarriage fetuses.

## Conclusion

AMA is a major risk factor for aneuploidy and CNVs. A better understanding of the types of aneuploidies and CNVs associated with maternal aging could lead to intervention strategies that may reduce their occurrence and prevent abnormal reproductive outcomes in the human population. In the present study, we revealed the characteristics of pathogenic CNVs, including mosaicism, microduplication/microdeletion, and LOH, in women of AMA. Our findings can aid clinicians to guide clinical decision-making and enable more informed clinical counseling for women of AMA.

## Data Availability

The raw data supporting the conclusion of this article will be made available by the authors, without undue reservation.
